# Anterior to Midposterior Corpus Callosum Subregions Are Volumetrically Reduced in Male Alcoholics but Only the Anterior Segment Is Associated to Alcohol Use

**DOI:** 10.3389/fpsyt.2019.00196

**Published:** 2019-04-05

**Authors:** Rodrigo Stênio Moll de Souza, Marcos Rosa Jr., Thayssa Dalla Costa Escobar, Emerson Leandro Gasparetto, Ester Miyuki Nakamura-Palacios

**Affiliations:** ^1^Department of Internal Medicine, Health Sciences Center, Federal University of Espírito Santo, Vitória, Brazil; ^2^University Hospital Cassiano Antônio de Moraes, Health Sciences Center, Federal University of Espírito Santo, Vitória, Brazil; ^3^Department of Radiology, Federal University of Rio de Janeiro, Rio de Janeiro, Brazil; ^4^BRAEN—Brazilian Research Group on Brain and Cognitive Engineering, Federal University of Espírito Santo, Vitória, Brazil; ^5^Laboratory of Cognitive Sciences and Neuropsychopharmacology, Graduation Program in Physiological Sciences, Federal University of Espírito Santo, Vitória, Brazil

**Keywords:** alcohol, magnetic resonance, volumetry, FreeSurfer, AUDIT

## Abstract

Alcohol consumption seems to affect corpus callosum morphometry irrespectively of an alcohol use disorder (AUD) diagnosis. The present study examined the relationship between corpus callosum (CC) subregion volumes and alcohol use patterns in AUD and non-AUD subjects. Twenty-two male AUD patients and 23 healthy matched non-AUD subjects were recruited from March 2016 to July 2017. Volumetric data were acquired through Magnetic Resonance and analyzed by the FreeSurfer software. AUD subjects were in abstinence for 45.1 days ± 36.8 (SD), consumed higher amounts of alcohol and presented higher AUDIT scores than controls (*p* < 0.0001). A multivariate analysis corrected by age and tobacco use indicated that AUD patients presented smaller CC volumes compared to non-AUD subjects (*p* < 0.01), except for the posterior subregion. A multiple regression analysis corrected by age and tobacco use including CC volumes from all subjects and the amount of daily alcohol ingestion as variables indicated that anterior CC volume was negatively (*p* < 0.001) associated to alcohol consumption. This study demonstrated that CC subregions were smaller in AUD subjects, as expected, and that the volume of the anterior segment was inversely associated to increasing daily amounts of alcohol, indicating greater frontal region vulnerability to harmful alcohol effects.

## Introduction

The corpus callosum (CC) is the largest commissure in the brain, constituted by massive white matter fibers connecting the left and right hemispheres, responsible for most of the communication between both sides of the brain (1).

Callosal morphometry alterations have been associated to neurodegenerative diseases and brain injury (2–6), including alcohol use disorder (AUD) (7, 8). Schulte et al. (1) demonstrated that white matter microstructure integrity throughout full CC extent (genu, body, and splenium) is abnormally low in AUD alcoholics when compared to age and gender matched controls, detected by diffusion tensor imaging (DTI). The authors suggest that white matter fiber coherence disruption contributes to mild but detectable disturbances in interhemispheric processing in alcoholism cases.

Changes in CC morphometry have also been reported in subjects who drink alcohol but do not present any history of alcohol abuse (9). Alcohol consumption in non-AUD subjects has been associated to CC volume in men, but not in women (9), suggesting that alcohol can be detrimental to CC integrity irrespectively of an established dependence.

Thus, considering that CC is altered in alcohol users and that specific structural segments may be related to long-term alcohol consumption, the aim of the present study was to investigate the association of CC subregion volumes segmented through the FreeSurfer software and the amount of ingested alcohol (drinks/day) by 45 male subjects, irrespectively of an alcohol dependence diagnosis. Segmented CC volumes between AUD patients and matched non-AUD controls were also compared.

## Methods

### Subjects

Twenty-two male patients diagnosed with AUD were successively recruited from a specialized public outpatient service belonging to the Espírito Santo Federal University Medical School Hospital (Brazil) from March 2016 to July 2017. Twenty-three healthy non-AUD age- and gender-matched subjects comprised the control group, recruited from relatives and workers presenting similar socio-demographic characteristics from the Espírito Santo Federal University Hospital.

The AUD group included: (i) male patients between the age of 30 and 70; (ii) the consumption of at least 30 drinks per week, on average, throughout the previous year; (iii) the presence of AUD criteria according to the ICD-10 and DSM-5; (iv) a stable clinical condition, with no need for inpatient care; (v) in alcohol abstinence for at least 15 days; (vi) able to read, write, and speak Portuguese; and (vii) without severe withdrawal signs or symptoms at baseline, (viii) accepting participation in the study and signing an informed consent form. Non-AUD subjects should be in accordance to items i, vi, and viii.

Exclusion criteria were as follows: (i) an intoxication or withdrawal condition due to a substance other than alcohol, (ii) the diagnosis of a neuropsychiatric or medical disorder other than alcohol dependence, except for nicotine and/or caffeine; (iii) a diagnosis of epilepsy, convulsions, or delirium tremens during alcohol abstinence; (iv) a previous history of drug hypersensitivity or adverse reactions to diazepam or other benzodiazepines, as well as haloperidol; (v) any contraindication regarding magnetic resonance procedures such as electronic implants, metal implants, claustrophobia, or permanent make-up, or any tattoo received within the previous 3 months; (vi) the presence of vascular, traumatic, inflammatory, or tumor injuries detectable by MRI examinations; (vii) a clinical or laboratorial diagnosis of active hepatic encephalopathy.

Ethical approval was provided by the Espírito Santo Federal University Brazilian Institutional Review Board (CAAE 19403713.6.0000.5060 and 13528213.2.0000.5060), Brazil. The study was carried out in strict adherence to the Declaration of Helsinki and in accordance with the ethical standards of the Committee on Human Experimentation of the Espírito Santo Federal University, ES, Brazil, where the study was conducted. Subjects were fully informed concerning the experimental protocol and voluntarily signed an informed consent form before the beginning of the study.

Subjects were clinically evaluated and interviewed regarding their socio-demographic characteristics and alcohol and drug use patterns, including an AUDIT application (Alcohol Use Disorder Identification Test), see description in the Supplementary Material (10).

### Magnetic Resonance Imaging (MRI)

Sagittal 3D T1-weighted images with a specific head coil model SENSE (8 channels) were acquired using a 1.5 T scanner (Philips Medical Systems Nederland B.V., The Netherlands), applying the following parameters: FOV: 240 × 240 mm; voxel size: 1.1 × 1.1 × 1.3 mm; flip angle: 12°; TR/TE: 20/4.8 ms; acquisition matrix: 220 × 220; 1.3 mm slice thickness with no gap, NSA: 2.

### FreeSurfer Post-processing

All images were transferred to a workstation (MacOS X Yosemite, version 10.10) with 16 GB of RAM memory and six Core Intel Xeon E5 3.5 GHz processors located in the Espírito Santo Federal University Radiology Sector, Brazil. Cortical segmentation and corrections after visual inspection for accuracy assessments were performed using the FreeSurfer version 5.3.0 software (http://surfer.nmr.mgh.harvard.edu) by two technicians, under the supervision of an onsite medical physicist and the online support of another medical physicist with 7 years of experience with this software. The corpus callosum (CC) was automatically segmented into five subregions, from genu to splenium, into anterior (CCAnterior), mid-anterior (CCMidAnterior), central (CCCentral), mid-posterior (CCMidPosterior), and posterior (CCPosterior) sections. The technical details of these procedures have previously been described (11–13).

### Statistical Analyses

Data are presented as percentages or means ± standard deviation (SD). CC subregion volumes, normalized by intracranial volume, were compared between groups (non-AUD vs. AUD) by a multivariate analysis using age (as this variable may influence CC volumes, as reported by Prendergast et al.) (14) and tobacco use (as this variable may influence CC volumes due to co-dependence) as covariates. Data were normally distributed according to the Shapiro–Wilk normality test, and a *p*-value lower than 0.01 was considered as statistically significant according to Bonferroni's multiple comparisons corrections considering five comparisons.

AUD and non-AUD CC subregion volumes were introduced as independent variables in multiple regression analyses corrected by age and tobacco use by the amount of alcohol ingested, as drinks/day (a drink was considered as roughly equivalent to 14 g of pure alcohol, independently of the type of the alcohol beverage). A two-tailed *p*-value of 0.05 was used to determine statistical significances for these assessments, as well as for inter-group age comparisons by an unpaired *t*-test and non-parametric data assessments by the Chi-square or Fisher tests.

The SPSS Statistics version 24 (IBM Corporation, USA) and Graph Pad Prism version 7 (GraphPad Software Inc., USA) were used for the statistical analyses and graphic presentations.

## Results

Age and tobacco use for both assessed groups, as well as drug use patterns in alcoholics, are presented in Table 1. Other socio-demographic characteristics are presented in the Supplementary Material.

**Table 1:** Age and tobacco use and alcohol use pattern in patients with Alcohol Use Disorder (AUD, *n* = 22) and controls (non-AUD, *n* = 23).

		**AUD (*n* = 22)**	**Non-AUD (*n* = 23)**		***p-*value**
Age [means (SD)] (min – max)		53.9 (8.8)	52.4 (9.9)	*t*_(43)_ = −0.54	0.59
		(30– 68)	(30–67)	–	–
Tobacco use	Yes	12 (54.5%)	6 (26.1%)	*X*_2_ = 5.0	0.08
*n* (%)	No	10 (45.5%)	15 (65.2%)		
**ALCOHOL USE**					
Amount of ingested alcohol (drinks/day) [mean (SD)]		14.7 (10.8)	0.22 (.52)	*t*_(21.1)_ = −6.4	<0.0001^****^
AUDIT [mean (SD)]		26.8 (7.4)	1.8 (2.6)	*t*_(25.8)_ = −15.3	<0.0001^****^
Age at alcohol use onset [mean (SD)]		15.9 (10.8)	–	–	–
Days of abstinence before the study [mean (SD)] (min–max)		45.1 (36.8)	–	–	–
		(15–181)			
Years of alcohol use [mean (SD)] (min–max)		38.1 (8.4)	–	–	–
		(15–54)			

*AUDIT, Alcohol Use Disorder Identification Test*,

**** *p < 0.0001*.

AUD patients and non-AUD controls were all males and matched by age, presenting means of 53.9 ± 8.8 (SD) and 52.4 ± 9.9 (SD) years old, respectively. An inter-group analysis regarding tobacco use indicated non-statistically significant differences, although a trend (*p* = 0.08) was observed (Table 1).

Non-AUD controls consumed an average of 0.22 ± 0.52 alcoholic drinks per day (19 out of 23 subjects, where 82.6%, were complete abstainers of alcohol use, and the remaining four consumed one to two drinks/day when ingesting alcohol). This amount was significantly lower (*p* < 0.0001) than that consumed by AUD patients, of 14.7 ± 10.8 (SD) drinks per day. In addition, AUDIT scores were significantly lower (*p* < 0.0001) in non-AUD subjects (mean of 1.8 ± 2.6 SD) compared to AUD controls (mean of 26.8 ± 7.4 SD), as expected (Table 1).

AUD patients began ingesting alcohol at mean age of 15.9 ± 10.8 (SD) years old, ingesting this substance for about 38.1 ± 8.4 (SD) years and were in abstinence for an average of 45.1 ± 36.8 (SD) (Table 1).

### Corpus Callosum Volume

#### Inter-group Comparisons

**Figure 1:**
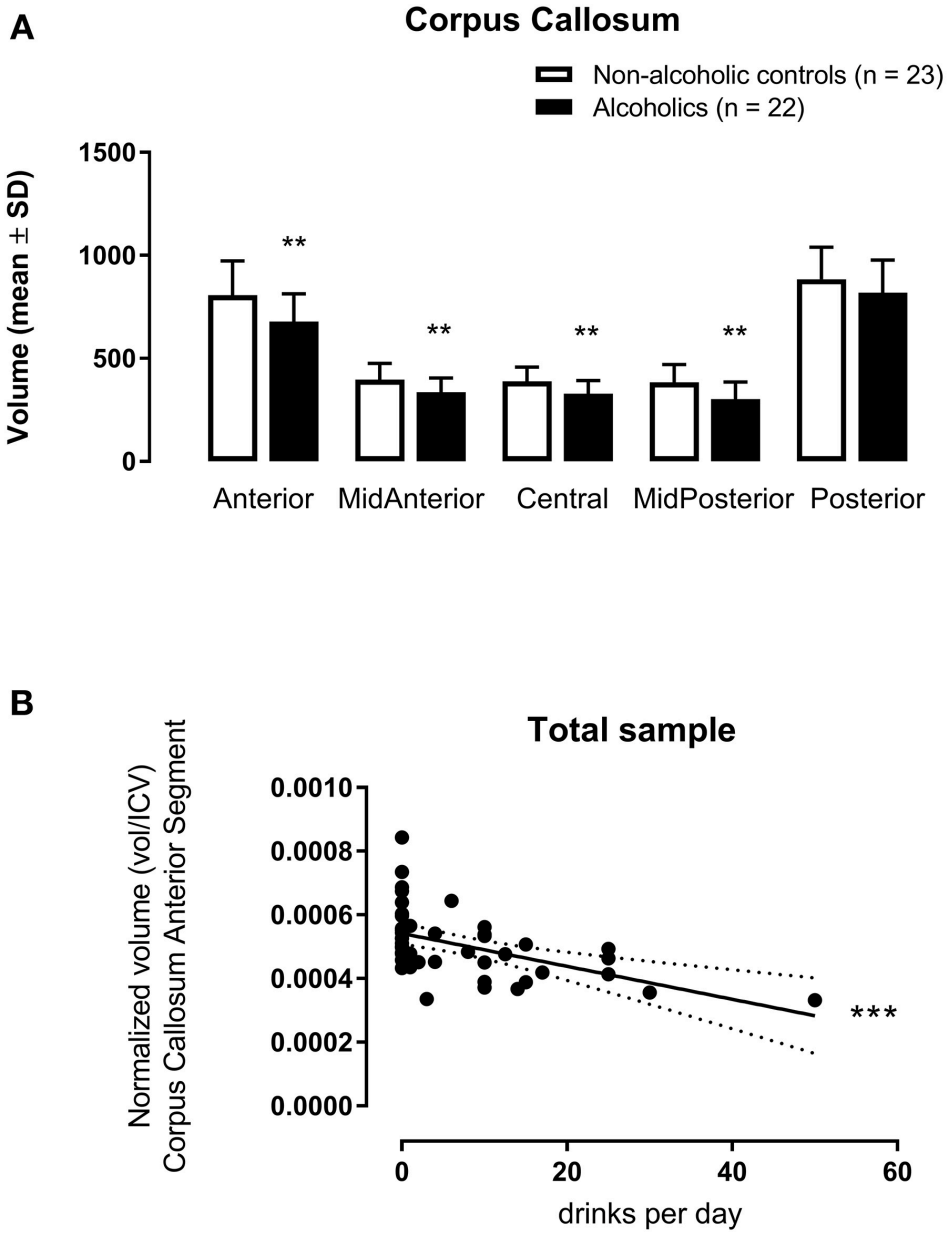
Corpus callosum subregion volumes (mm^3^) [anterior (CCAnterior), mid-anterior (CCMidAnterior), central (CCCentral), mid-posterior (CCMidPosterior), and posterior (CCPosterior)] normalized by intracranial volume (icv in mm^3^) in 22 alcohol use disorder (AUD) subjects and 23 matched non-AUD controls. Segmentation was performed by the automatic reconstruction of the cerebral cortex from magnetic resonance imaging data acquired in high-resolution T1 by the FreeSurfer software package. **(A)** **p < 0.01 (Multivariate Analysis corrected by age and tobacco use); **(B)** ***p < 0.001 (Multiple Linear Regression Analysis corrected by age and tobacco use).

The multivariate analysis corrected by age and tobacco use indicated significant inter-groups differences concerning CCAnterior [*F*_(1, 41)_ = 11.23, *p* = 0.002, η^2^ = 0.215], CCMidAnterior [*F*_(1, 41)_ = 9.65, *p* = 0.003, η^2^ = 0.191], CCCentral [*F*_(1, 41)_ = 8.91, *p* = 0.005, η^2^ = 0.179], and CCMidPosterior [*F*_(1, 41)_ = 9.78, *p* = 0.003, η^2^ = 0.193] volumes (Figure 1A). The only segment with no difference between AUD and non-AUD subjects was the CCPosterior [*F*_(1, 41)_ = 3.27, *p* = 0.078, η^2^ = 0.074].

#### Multiple Linear Regressions

When considering alcohol drinks per day as the dependent variable and the five CC segments as independent variables, with age and tobacco use as covariables for all subjects, CCAnterior volume accounted for 23.1% of the data variance for this variable, *F*_(3, 44)_ = 5.402, *p* = 0.003, adjusted *R*^2^ = 0.231, 95% CI [−74.99, −21.40]. This CC subregion displayed a significant zero-order correlation (*r* = −0.508, β = −0.491) to drinks/day (Figure 1B), with a significant (*p* = 0.0002) partial effect in the full model.

Considering that the mean age was equivalent in both groups, the inter-groups and regression findings remain the same when not controlling for this variable.

## Discussion

Volumes were significantly reduced in the segmented CC subregions, except for the posterior segment in AUD patients when compared to well-paired non-AUD subjects, as expected (7, 8).

According to Goldman et al. (3), regional callosal atrophy in Parkinson's disease is predictive of cognitive domain performance, with central volumes associated to the attention/working memory domain and midposterior volumes, to the executive function, language, and memory domains.

However, when considering all subjects, both AUD and non-AUD, included in the present study, the anterior CC segment volume displayed a negative association to alcohol consumption, determined as drinks per day. Thus, the higher the amount of ingested alcohol, the smaller the anterior CC volume.

Accordingly, studies relating alcohol use and CC morphometry have indicated important changes in frontal CC segments. Kapogiannis et al. (9) demonstrated that higher alcohol consumption in non-AUD subjects was associated to smaller premotor frontal CC volume in male subjects. Liu et al. (8) reported that the segment interconnecting the bilateral orbitofrontal cortices was the most affected, and that it was related to impulsivity levels. DTI studies have indicated reduced diffusion anisotropy in the CC genu and centrum semiovale (15), in frontal sites such as the frontal forceps, internal and external capsules and fornix (16), i.e., within the frontal white matter of chronic AUD patients (17).

According to Pfefferbaum et al. (16), frontal and superior sites present the greatest abnormalities in AUD patients compared to non-AUD subjects, while more posterior and inferior bundles are relatively preserved. In the present study, the volume of the posterior CC, which connects occipital, temporal and parietal regions (18), was also quite preserved, as no difference was noted compared to non-AUD subjects, while the anterior CC was significantly smaller and related to the amount of alcohol consumption.

In a DTI study, Harris et al. (17) demonstrated dysfunctions related to AUD in white matter tracts connecting the frontal lobe and cingulate regions of the cortical-subcortical brain reward circuitry. This suggests damage to fronto-limbic connections at the microstructural level, especially in the right hemisphere, associated to visuospatial and emotional abnormalities, as well as to working memory deficits, commonly seen in AUD subjects. The results reported herein corroborate this evidence regarding the frontal region, indicating greater vulnerability to alcohol effects, since the anterior (or genu) CC, which interconnects the lateral and medial frontal regions between the right and left hemispheres, was the only segment related to amount of alcohol consumption irrespectively of an alcohol dependence diagnosis.

In addition to most socio-demographic characteristics, the assessed groups in the present study were also well-matched regarding mini-mental status examination performance when regularly screened in the outpatient service (see Supplementary Material for more details), but were not examined regarding more complex cognitive and executive tasks, due to time restrictions in their routine assessments. This limitation prevented the examination of the potential relationship between CC subregion volume and cognitive performance. Another important limitation was the fact that only male subjects were included in the study, as most AUD patients attended in our public outpatient service are male. Thus, in order to include females in a representative manner, different outpatient services with different treatment approaches would have to be involved.

In summary, CC segment volumes were significantly lower in AUD patients when compared to non-dependent healthy controls, with the exception of the posterior subregion. In addition, anterior segment volume was associated to increasing daily alcohol use, possibly indicating greater frontal region vulnerability to harmful alcohol effects.

## Ethics Statement

Ethical approval was provided by the Brazilian Institutional Review Board of the Federal University of Espírito Santo (CAAE 19403713.6.0000.5060 and 13528213.2.0000.5060), Brazil.

## Author Contributions

RdS, MR, and EN-P conceived of the presented idea and contributed with important theoretical and technical content. RdS coordinated the recruitment of patients, established the parameters, and scheduled the MRI acquisition. TE contributed with image data post-processing. EN-P supervised the study, run data analysis, and organized the manuscript. EG contributed with technical knowledge and co-supervised the work. All authors discussed the results and contributed to the final manuscript.

### Conflict of Interest Statement

The authors declare that the research was conducted in the absence of any commercial or financial relationships that could be construed as a potential conflict of interest.
