# Supplementary material for: Anterior to Midposterior Corpus Callosum Subregions Are Volumetrically Reduced in Male Alcoholics but Only the Anterior Segment Is Associated to Alcohol Use
Source: Front Psychiatry. 2019 Apr 5;10:196. doi: 10.3389/fpsyt.2019.00196 (PMC6460345; doi:10.3389/fpsyt.2019.00196)
Supplement: Supplementary file 1 [file Data_Sheet_1.docx]

***Supplementary material***

***Clinical assessments***

*Alcohol Use Disorder Identification Test (AUDIT)*

This is a simple method of screening for excessive drinking and to assist in brief assessment (1). It is composed by 10 items, each one ranging from 0 to 4, with maximum score of 40. Total score of 8 or more is indicative of hazardous and harmful alcohol use, as well as possible alcohol dependence. Scores from 8 to 15 would represent a medium level of alcohol problems and scores of 16 and above represent a high level of alcohol problems (1). Four levels of risk drinking can be considered: Zone I (between 0 and 7) would be of low risk drinking or abstinence; Zone II (between 8 and 15), would consist of alcohol use in excess, requiring brief intervention using simple advice and education materials; Zone III, ranging from 16 to 19, would be harmful and hazardous drinking and can still be managed by a combination of simple advice, brief counseling and continued monitoring, but Zone IV, above 20, would indicate alcohol dependence, and patients would require to be referred to a specialist for diagnostic evaluation and possible treatment (1).

*Mini Mental Status Examination (MMSE)*

An adapted version of the MMSE in Portuguese was used. This version included an 11-item examination that examined five areas of cognitive function: orientation, registration, attention and calculation, recall, and language. The maximum score that could be achieved was 30, while a mean score between 23 and 28 would be expected according to the age and educational level of our alcoholic and non-alcoholic subjects (2).

***References***

1. Barbor TF, Higgins-Biddle JC, Saunders JB, Monteiro MG. *AUDIT: The Alcohol Use Disorders Identification Test. Guidelines for Use in Primary Care.* Second ed. Geneva, Switzerland: World Health Organization.Department of Mental Health and Substance Dependence (2001). 41 p.

2. Crum RM, Anthony JC, Bassett SS, Folstein MF. Population-based norms for the Mini-Mental State Examination by age and educational level. *JAMA* (1993) 269(18):2386-91. PubMed PMID: 8479064.

Table S1. Socio-demographic characteristics in patients with Alcohol Use Disorder (AUD, n = 22) and control (non-AUD, n = 23).

|  | **Non-AUD**  **(n = 23)** | | **AUD**  **(n = 22)** |  | ***p value*** |
| --- | --- | --- | --- | --- | --- |
| **Socio-demographic characteristics and cognitive performance** | | | | | |
| Gender *n* (*%*) | Male | 23 (100%) | 22 (100%) |  |  |
| Years of education  *n* (*%*) | Up to 5  Between 6 to 9  Between 10 to 13  Between 14 to 19 | 6 (26.1%)  9 (39.1%)  7 (30.4%)  1 (4.3%) | 10 (45.5%)  8 (36.4%)  4 (18.2%)  0 (0.0%) | X_2_ = 2.9 | .41 |
| Employment situation  *n* (*%*) | Formal job  Informal job  Unemployed  Retired  Freelance  Not reported | 14 (60.9%)  5 (21.7%)  0 (0.0%)  3 (13.0%)  1 (4.3%)  0 (0.0%) | 1 (4.5%)  2 (9.1%)  9 (40.9%)  3 (13.6%)  5 (22.7%)  1 (4.5%) | X_2_ = 26.2 | .0002*** |
| Marital state  *n* (*%*) | Single  Married or common-law marriage  Divorced  Not reported | 5 (21.7%)  16 (69.6%)  1 (4.3%)  1 (4.3%) | 5 (22.7%)  11 (50.0%)  6 (27.3%)  0 (0.0%) | X_2_ = 5.5 | .14 |
| MMSE  *[mean (SD*)] |  | 27.0 (2.6) | 26.3 (2.7) | F(1,41) = .014 | .91 |

*** p < 0.001. They were all males, and except for employment situation, no other characteristics, such as schooling and marital state differed from non-AUD control group. MMSE: Mini Mental Status Examination (between-groups analysis was performed by a univariate analysis with age and tobacco use as covariates as these variables could influence the cognitive performance).
